# Partial decellularization eliminates immunogenicity in tracheal allografts

**DOI:** 10.1002/btm2.10525

**Published:** 2023-04-21

**Authors:** Zheng Hong Tan, Lumei Liu, Sayali Dharmadhikari, Kimberly M. Shontz, Lily Kreber, Sarah Sperber, Jane Yu, Woo Yul Byun, Sarah C. Nyirjesy, Amy Manning, Susan D. Reynolds, Tendy Chiang

**Affiliations:** ^1^ Center of Regenerative Medicine, Abigail Wexner Research Institute, Nationwide Children's Hospital Columbus Ohio USA; ^2^ College of Medicine, The Ohio State University Columbus Ohio USA; ^3^ Department of Pediatric Otolaryngology Nationwide Children's Hospital Columbus Ohio USA; ^4^ Center for Perinatal Research, Nationwide Children's Hospital Columbus Ohio USA

**Keywords:** decellularization, immunogenicity, orthotopic tracheal transplantation, regenerative medicine, tissue‐engineered tracheal graft

## Abstract

There is currently no suitable autologous tissue to bridge large tracheal defects. As a result, no standard of care exists for long‐segment tracheal reconstruction. Tissue engineering has the potential to create a scaffold from allografts or xenografts that can support neotissue regeneration identical to the native trachea. Recent advances in tissue engineering have led to the idea of partial decellularization that allows for the creation of tracheal scaffolds that supports tracheal epithelial formation while preserving mechanical properties. However, the ability of partial decellularization to eliminate graft immunogenicity remains unknown, and understanding the immunogenic properties of partially decellularized tracheal grafts (PDTG) is a critical step toward clinical translation. Here, we determined that tracheal allograft immunogenicity results in epithelial cell sloughing and replacement with dysplastic columnar epithelium and that partial decellularization creates grafts that are able to support an epithelium without histologic signs of rejection. Moreover, allograft implantation elicits CD8+ T‐cell infiltration, a mediator of rejection, while PDTG did not. Hence, we establish that partial decellularization eliminates allograft immunogenicity while creating a scaffold for implantation that can support spatially appropriate airway regeneration.

## 1 INTRODUCTION

Tracheal defects are rare but life‐threatening. End‐to‐end anastomosis is not feasible beyond a certain length, warranting tissue replacement for reconstruction.^1^ Unfortunately, there is no clinical standard for tracheal replacement. Creation of a graft that can recapitulate the complex structure and function of the trachea is a priority within the field of regenerative medicine.

Decellularization is a proven method of creating non‐immunogenic scaffolds via regenerative medicine, with several United States Food and Drug Administration approved applications.^2^, ^3^, ^4^, ^5^ When applied to the trachea, complete decellularization has formed scaffolds capable of supporting functional neotissue formation in vitro, but in vivo performance is limited by the loss of mechanical properties.^6^, ^7^, ^8^, ^9^, ^10^, ^11^, ^12^ Tracheal cartilage provides the primary structural support for the organ and efforts to decellularize chondrocytes from the dense extracellular matrix result in a loss of graft patency.^9^, ^10^ However, complete decellularization may not be necessary given the immunoprivileged location of chondrocytes within the dense cartilage extracellular matrix.^8^, ^13^, ^14^, ^15^ Advances in tracheal tissue engineering have relied upon partial decellularization or de‐epithelialization, resulting in removal of the cells within the tracheal epithelium with preservation of graft chondrocytes.^15^, ^16^

Using a murine model of orthotopic tracheal transplant, we previously established that partially decellularized tracheal grafts (PDTG) are capable of tracheal neotissue formation with preservation of mechanical properties.^16^, ^17^ Still, the ability of partial decellularization to eliminate graft immunogenicity remains unknown, and understanding the immunogenic properties of PDTG is a critical step toward clinical translation. We use our murine microsurgical model to determine if partial decellularization eliminates allograft immunogenicity, and assess the impact of immunogenicity.

## 2 METHODS

### 2.1 Animal care and ethics statement

The Institutional Animal Care and Use Committee of the Abigail Wexner Research Institute at Nationwide Children's Hospital (Columbus, OH) reviewed and approved the protocol (AR15‐00090). All animals received humane care by standards published by the Public Health Service, National Institutes of Health (Bethesda, MD) in the Care and Use of Laboratory Animals (2011), and US Department of Agriculture (USDA) regulations outlined in the Animal Welfare Act.

### 2.2 Fabrication of PDTGs

Tracheal grafts were harvested from female 6 to 8‐week‐old C57BL/6 and Balb/c mice as previously published.^8^, ^18^ Proximal tracheas were dissected, and a 5 mm tracheal segment was harvested and cryopreserved at −80°C in 1 mL cryopreservation solution (Dulbecco's Modified Eagle Medium [ATCC, Manassas, VA] with 10% fetal bovine serum, 1% Penicillin/streptomycin, and 5% Dimethyl sulfoxide [DMSO, ATCC]).^19^

PDTG derived from Balb/c (partially decellularized tracheal allografts [PDTA]) and from C7BL/6 (partially decellularized tracheal syngrafts [PDTS]) were produced with a 7‐h decellularization protocol. Briefly, tracheas were rinsed with 1× PBS with 1% penicillin/streptomycin (P/S, Gibco, Thermo Fisher Scientific, Waltham, MA) and treated with 0.01% sodium dodecyl sulfate (SDS, Sigma–Aldrich, MO) and 0.9% sodium chloride (NaCl, Fisher Scientific, Fair Lawn, NJ) for 5 min. The tracheas were then subjected to consecutive 3 h graded SDS treatments of 0.01% and 0.1% SDS before soaking in 0.2% SDS and 0.1% SDS for 15 min each. They were then treated with 1% Triton X‐100 in distilled water for 5 min to remove nucleic acids and underwent a final 0.9% NaCl wash for 15 min. All steps were performed on a shaker at the speed of 48 rounds/min. PDTG were cryopreserved at −80°C until use.

### 2.3 Implantation of tracheal grafts

Syngeneic tracheal grafts (STG), PDTA, PDTS, and allografts (ATG) were implanted via previously published methods (*N* = 5/STG, *N* = 10/PDTA, PDTS, and ATG).^8^, ^18^ A 5 mm tracheal segment was harvested and immersed in phosphate‐buffered saline for implantation. At the time of implantation, 4 mm segment was resected and orthotopically implanted.^8^ Type of grafts implanted were randomized within blocks of “day of procedure” and “surgeon” to experimental groups and euthanized at various timepoints. Only female mice were used in this study to avoid sex‐specific host responses to implanted grafts.^20^ 10 days was used as a timepoint for acute rejection and at 1‐ and 3‐months were used for chronic rejection timepoints.^21^, ^22^, ^23^ Animals were closely monitored for early (humane) euthanasia criteria including respiratory distress (labored breathing, stridor) and/or more than 20% weight loss compared to weight before surgery. At a planned or humane endpoint, animals were euthanized with Ketamine/Xylazine cocktail. Once euthanasia was confirmed, the entire trachea including the graft was harvested and placed in 10% Neutral buffered formalin.

### 2.4 Histology

Formalin‐fixed STG, PDTA, PDTS, and allografts were decalcified in 15% EDTA at 4°C overnight before being paraffin‐embedded. Longitudinal sections (4 μm) were then sectioned with microtome. The sections were then de‐paraffinized with xylenes, rehydrated with decreasing concentrations of ethanol, and stained with hematoxylin (Sigma–Aldrich, MO) and counterstained with eosin. Collagen fibers of tracheal grafts implantation were stained with Masson's Trichrome. Apoptotic cells were identified using the Terminal deoxynucleotidyl transferase dUTP nick end labeling assay (TUNEL).

#### 2.4.1 T‐cell infiltration

T‐cell infiltration was assessed via immunofluorescent staining of CD4 and CD8 T‐cells. Briefly, the sections were stained with Rabbit Anti‐CD4 (1:500 dilution) and Rabbit Anti‐CD8 (1:250 dilution) and Anti‐rabbit Alexa Fluor 594 as the secondary antibody. T‐cell infiltration was assessed by quantifying the number of T‐cells per submucosal area.

#### 2.4.2 Epithelial height

Epithelialization was assessed with hematoxylin and eosin (H&E) and immunofluorescent staining of post‐implantation tracheal sections. Images of stained sections were captured using bright field and immunofluorescent microscopy (Zeiss, Oberkochen, Germany). Average epithelial height was measured by dividing the area of the graft epithelium by the length of the graft basement membrane.

#### 2.4.3 Epithelialization

Longitudinal sections were stained with Mouse anti‐Acyl Alpha‐tubulin (ACT) to identify ciliated epithelial cells. The extent of epithelial infiltration was measured by dividing percent coverage of the ACT‐positive cells by the length of the graft.^18^

#### 2.4.4 Submucosal thickness

Submucosal thickness was quantified using ImageJ software (U. S. National Institutes of Health, Bethesda, MD) and calculated by averaging five regularly spaced measurements of the submucosal height between the cartilage and basement membrane on each graft cartilage ring.

#### 2.4.5 Micro‐computed tomography

Micro‐computed tomography (microCT) imaging was performed on live animals to assess graft patency at 1 month and 3 months using a μPET/CT system (U‐PET6CTHR, MILabs, Utrecht, The Netherlands). The animals were anesthetized with inhalational isoflurane in room air at 1–3 L/min and positioned prone. The scan was set as full 360° rotation, x‐ray tube of 0.33 mA and 55 kV, 0.750° per step, 1 projection per step, 1 × 1 binning, and 40 ms exposure time. All microCT images were reconstructed using MILabs reconstruction software v12.0 (Utrecht, The Netherlands) with a 40 μm voxel grid, Hann projection filter, and Gaussian volume filter (160 μm). The area of airway lumen was quantified from each slice of graft scans and analyzed using ImageJ software.

#### 2.4.6 Statistical analysis

Normally distributed data were compared using Welch's *t*‐test for data with non‐equal variances and unpaired *t*‐test and ANOVA for data with equal variances. Non‐parametric tests (Mann Whitney‐*U*) were used for data that were not distributed normally. Statistical tests were performed using the GraphPad Prism 8 software (GraphPad Software Inc., CA). Statistical difference was defined as *p* < 0.05. Experimental data were expressed as means ± standard deviations (SD).

## 3 RESULTS

### 3.1 Orthotopic tracheal transplantation resulted in similar graft patency and survival among graft types

**FIGURE 1:**
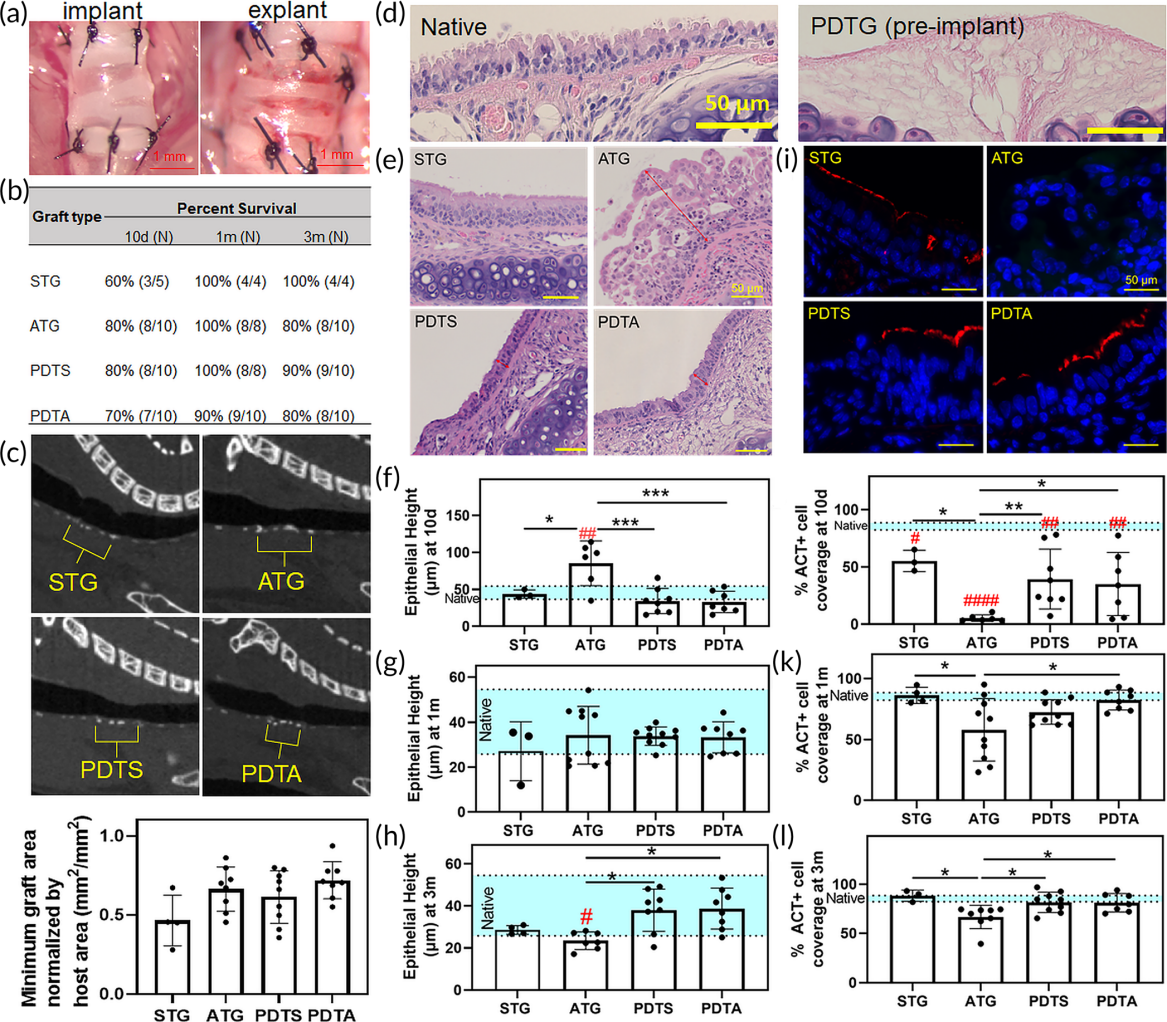
Orthotopic tracheal transplantation resulted in similar graft patency and survival among graft types. (a) Representative images of orthotopic tracheal transplantation. (b) Orthotopic tracheal transplantation survival rates. Animals were euthanized at 10 days, 30 days, and 90 days for end‐point analysis. (c) Sagittal micro‐computed tomography of the airway demonstrating graft patency, quantification of graft cross‐sectional area (yellow brackets denote graft). (d) Representative H&E sections of native and PDTG (preimplant). (e) Representative longitudinal H&E sections of tracheal grafts (10 days). (f) Epithelial height (10 days, # represents increased epithelial height in Allograft [ATG] compared to native, * = increased epithelial height in Allografts vs other graft types [^##^ *p* = 0.0063 vs. native, *p = 0.028 vs. STG, ***p = 0.0003 vs. PDTA, ***p = 0.0003 vs. PDTS]). (g) Epithelial height at 1‐month. (h) Epithelial height at 3‐month # = decreased epithelial height in Allograft versus native (*p* = 0.0493) * = decreased epithelial height in Allografts compared to other graft types (*p* = 0.0143 ATG vs. PDTA, *p* = 0.0211 Allograft vs. PDTS). (i) Representative IF images of tracheal epithelium and multiciliated cells (ACT+ [red], 10 days). (j) % Graft coverage with ACT+ epithelial cells (10 days, # = decreased epithelialization vs native, *p* = 0.0003 vs. STG, *p* = 0.0037 vs. PDTS, *p* = 0.0019 vs. PDTA, *p* < 0.0001 vs. Allograft, * = change in epithelialization within graft types, *p* = 0.0238 Allograft vs. STG, *p* = 0.0013 ATG vs. PDTS, *p* = 0.0221 Allograft vs. PDTA). (k) % ACT cell coverage at 1‐month, * = decreased epithelialization versus other graft types (*p* = 0.0313 Allograft vs. STG, *p* = 0.0178 PDTA vs. Allograft). (l)% ACT cell coverage at 3‐month, * = decreased epithelialization compared versus graft types (*p* = 0.0242 STG vs. Allograft, *p* = 0.0402 PDTA vs. ATG, *p* = 0.0241 PDTS vs. Allograft). H&E, hematoxylin and eosin; IF, immunofluorescent; PDTA, partially decellularized tracheal allografts; PDTG, partially decellularized tracheal graft; PDTS, partially decellularized tracheal syngrafts; STG, syngeneic tracheal grafts.

All graft types remained patent with no evidence of stenosis and had similar survival rates (Figure 1a–c). The animals tolerated orthotopic tracheal transplantation well and did not exhibit signs of respiratory distress at the time of euthanasia. At Day 10, allografts were found to have diffuse epithelial sloughing and eosinophilic cellular infiltrate within the lamina propria consistent with acute rejection. This process also resulted in an increase in epithelial height and the loss of ciliated cells (Figure 1d–l; Figure S1).^21^, ^24^, ^25^ There was no sign of epithelial injury or eosinophilic infiltrate in surgical control (STG), which maintained an epithelium morphologically identical to the native trachea (Figure S1). PDTG also lacked signs of injury and eosinophilic cell infiltration, exhibiting early graft epithelialization. Terminally differentiated ciliated cells were seen repopulating PDTG at Day 10 and recreated pseudostratified epithelium by 1‐month (Figure 1i–l; Figure S2). Conversely, allografts demonstrated a blunted epithelium with less ciliation (*p* = 0.0149) (Figure 1j–l). There was no difference in epithelial morphology between allograft‐derived (PDTA) and syngeneic‐derived (PDTS) partially decellularized grafts (Figure 1d–l; Figures S2 and S3).

### 3.2 Partial decellularization attenuates CD8+ T‐cell mediated rejection

**FIGURE 2:**
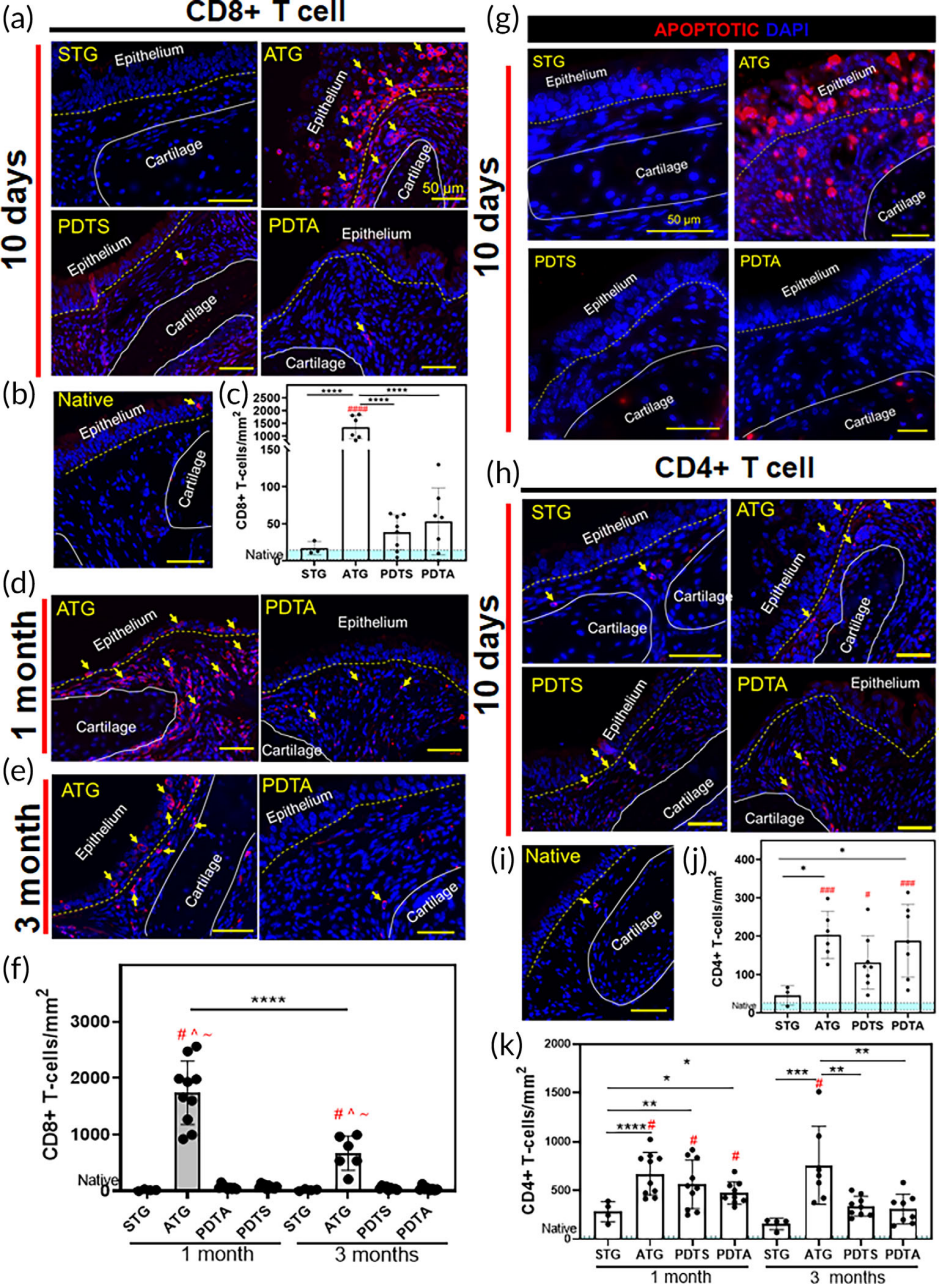
Partial decellularization attenuates CD8+ T‐cell mediated rejection. (a) Representative IF images of CD8+ T‐cells in grafts at 10 days and (b) native trachea (c) CD8+ T‐cells/mm^2^ (10 days) #### = increased CD8+ T‐cells vs Native (10 days, *p* < 0.0001) **** = decreased CD8+ T‐cells compared to Allograft (ATG) (10 days, *p* < 0.0001 for all graft types) * = decreased CD8+ T‐cells compared to Allograft at 10 days (*p* = 0.0324 STG vs. PDTA, *p* = 0.0184 STG vs. Allograft). (d) Representative images of CD8+ T‐cells in grafts at 1‐month and (e) 3‐months. (f) CD8+ T‐cells/mm^2^ at 1‐month and 3‐months # = increased CD8+ T‐cells versus STG (*p* < 0.0001 for STG‐1 m vs. Allograft‐1 m, *p* < 0.0001 for STG‐3 m vs. ATG‐3 m), ^ = increased CD8+ T‐cells versus PDTA (*p* < 0.0001 for PDTA‐1 m vs. Allograft‐1 m, *p* < 0.0001 for PDTA‐3 m vs. Allograft‐3 m), ~ = increased CD8+ T‐cells versus PDTS (*p* < 0.0001 for PDTS‐1 m vs. Allograft‐1 m, *p* < 0.0001 for PDTS‐3 m vs. Allograft‐3 m), **** = decreased CD8+ T‐cells between Allograft at 1‐ and 3‐month (*p* < 0.0001). (g) Representative Terminal deoxynucleotidyl transferase dUTP nick end labeling assay (TUNEL) images of the implanted grafts. red denotes apoptotic cells while blue denotes cellular nuclei. (h) Representative IF images of CD4+ T‐cells in grafts at 10 days and (i) native trachea. (j) CD4+ T‐cells/mm^2^ at 10 days # = increased CD4+ T‐cells versus Native at 10 days (*p* = 0.0242 vs. PDTS, *p* = 0.0007 vs. PDTA, *p* = 0.0004 vs. Allograft). (k) CD4+ T‐cells/mm^2^ at chronic time points * = increase in CD4+ T‐cells between grafts (*p* = 0.0312 for STG 1 m vs. PDTA 1 m, *p* = 0.0014 for STG 1 m vs. PDTS 1 m, *p* < 0.0001 for STG 1 m vs. Allograft 1 m, *p* = 0.0004 for STG 3 m vs. Allograft 3 m, *p* = 0.0011 for PDTA 3 m vs. Allograft 3 m, *p* = 0.0020 for PDTS 3 m vs. Allograft 3 m, *p* = 0.0066 for STG 1 m vs. STG 3 m, *p* = 0.0219 for PDTA 1 m vs. PDTA 3 m, *p* = 0.0226 for PDTS 1 m vs. PDTS 3 m). IF, immunofluorescent; PDTA, partially decellularized tracheal allografts; PDTS, partially decellularized tracheal syngrafts; STG, syngeneic tracheal grafts.

We measured T‐cell infiltration during acute (Day 10) and chronic (1, 3 months) intervals to quantify the immunogenicity of PDTG. In allografts, there was both acute and chronic elevation of CD8+ T‐cells within lamina propria (Figure 2a–f). Large amounts of CD8+ T‐cells in allografts were associated with apoptotic cells, both of which were not found STG and PDTG (Figure 2g). CD8+ T‐cells were found to be similar in PDTA and PDTS, suggesting that partial decellularization eliminated allograft immunogenicity. In both PDTG and allografts, CD4+ T‐cells increased at 10 days and 1 month (Figure 2h–k). At 3 months, CD4+ T‐cells in PDTG were found to be equivalent to control, while allograft CD4+ T‐cells remained persistently elevated.

## 4 DISCUSSION

Using a mouse microsurgical model, we determined that tracheal graft immunogenicity results in epithelial cell sloughing and replacement with dysplastic columnar epithelium characterized by both epithelial height and ciliated cell coverage of the graft.^21^, ^24^, ^25^ Presence of CD4+ and CD8+ T‐cells was not associated with stenosis. Epithelial changes in allografts also appeared to be associated with an increase in CD8+ T cells, an established mediator of rejection.^26^, ^27^, ^28^ Furthermore, partial decellularization removed graft immunogenicity, with no differences between PDTA and PDTS neotissue formation, and CD8+ T‐cell levels are similar to controls and baseline. Since PDTA is generated from an allogenic trachea, this suggests that partial decellularization does not result in acute or chronic rejection.

Our findings also suggest that neo‐epithelialization is at least partially mediated by CD4+ T‐cells. Some CD4+ T‐cells phenotypes such as T‐regulatory cells are essential for tissue repair and regeneration.^29^, ^30^ While the increased CD4+ T‐cell presence in allografts could be attributed to rejection, the elevated CD4+ T‐cell levels in PDTG at 1 month with a subsequent decrease at 3 months after ciliated epithelial regeneration, suggests that CD4+ presence is associated with neotissue formation. This is further supported by the baseline levels of CD4+ T‐cells in STG, which serve as a control for tracheal replacement.

One limitation of this study is the inability to use the well‐established method of flow cytometry to assess alloreactivity and T‐cell phenotypes due to the prohibitive size of the mouse trachea. In summary, we have established the potential of partial decellularization to eliminate the immunogenicity of tracheal allografts while creating a scaffold for implantation that can support spatially appropriate airway regeneration.

## 5 CONCLUSION

We have established that partial decellularization creates grafts that are able to support epithelization while remaining patent in vivo with similar survival rates to surgical controls. Moreover, partial decellularization does not result in rejection, indicating its potential to eliminate the immunogenicity of tracheal allografts.

## AUTHOR CONTRIBUTIONS

**Zheng Hong Tan:** Conceptualization (equal); data curation (lead); formal analysis (lead); investigation (lead); methodology (lead); validation (lead); visualization (lead); writing – original draft (lead); writing – review and editing (lead). **Lumei Liu:** Conceptualization (supporting); data curation (equal); formal analysis (equal); investigation (equal); methodology (equal); supervision (equal); writing – original draft (supporting); writing – review and editing (equal). **Sayali Dharmadhikari:** Formal analysis (equal); investigation (equal); methodology (equal); validation (equal); visualization (equal); writing – review and editing (equal). **Kimberly M. Shontz:** Investigation (equal); methodology (equal); project administration (equal); validation (equal); visualization (equal); writing – review and editing (equal). **Lily Kreber:** Formal analysis (equal); investigation (equal); methodology (equal); validation (equal); visualization (equal); writing – review and editing (equal). **Sarah Sperber:** Investigation (equal); methodology (equal); validation (equal); visualization (equal); writing – review and editing (equal). **Jane Yu:** Investigation (equal); methodology (equal); validation (equal); visualization (equal); writing – review and editing (equal). **Woo Yul Byun:** Investigation (equal); validation (equal); visualization (equal); writing – review and editing (equal). **Sarah C. Nyirjesy:** Investigation (equal); methodology (equal); validation (equal); writing – review and editing (equal). **Amy Manning:** Investigation (equal); methodology (equal); validation (equal); writing – review and editing (equal). **Susan D. Reynolds:** Data curation (equal); investigation (equal); methodology (equal); resources (equal); supervision (equal); writing – review and editing (equal). **Tendy Chiang:** Conceptualization (equal); data curation (equal); formal analysis (equal); funding acquisition (equal); investigation (equal); methodology (equal); project administration (equal); resources (equal); supervision (lead); validation (equal); visualization (equal); writing – review and editing (equal).

## FUNDING INFORMATION

The work presented was funded by NIH NHLBI K08HL138460 and R01HL157039 (Tendy Chiang is the recipient).

## CONFLICT OF INTEREST STATEMENT

The authors have no conflicts of interest to disclose as described by the American Journal of Transplantation.

### PEER REVIEW

The peer review history for this article is available at https://www.webofscience.com/api/gateway/wos/peer-review/10.1002/btm2.10525.

## Supporting information


**Figure S1:** Tracheal allograft presents with epithelial sloughing and eosinophilic infiltrates. Representative images of native trachea and tracheal grafts at 10 days post‐implantation. Native trachea and STG present with a columnar pseudostratified epithelium while ATG present with epithelial sloughing and eosinophilic infiltrates denoted by ▼. PDTS and PDTA presents with ciliated neo‐epithelium repopulating the graft.

**Figure S2:** Representative IF images of epithelization (ACT+) for the grafts at 1‐ and 3‐months post implant. Green denotes terminally differentiated ciliated cells (FoxJ1) while red denotes ciliated epithelium (ACT). PDTA and PDTS had similar epithelialization compared to STGs while ATGs had less ciliated epithelium.

**Figure S3:** Representative Masson Trichrome stains of PDTA and PDTS at 1 m. Blue indicates collagen deposition. Similar amounts of collagen were observed between PDTA and PDTS, indicating that they are likely to have similar amounts of collagen deposition and no fibrosis as a result from rejection is occurring.

## Data Availability

The data that support the results of this study, experimental protocols, and additional details regarding methods employed in this study will be made available through request with the corresponding author.
