# Supplementary figures and images for: Partial decellularization eliminates immunogenicity in tracheal allografts
Source: Bioeng Transl Med. 2023 Apr 21;8(5):e10525. doi: 10.1002/btm2.10525 (PMC10487308; doi:10.1002/btm2.10525)

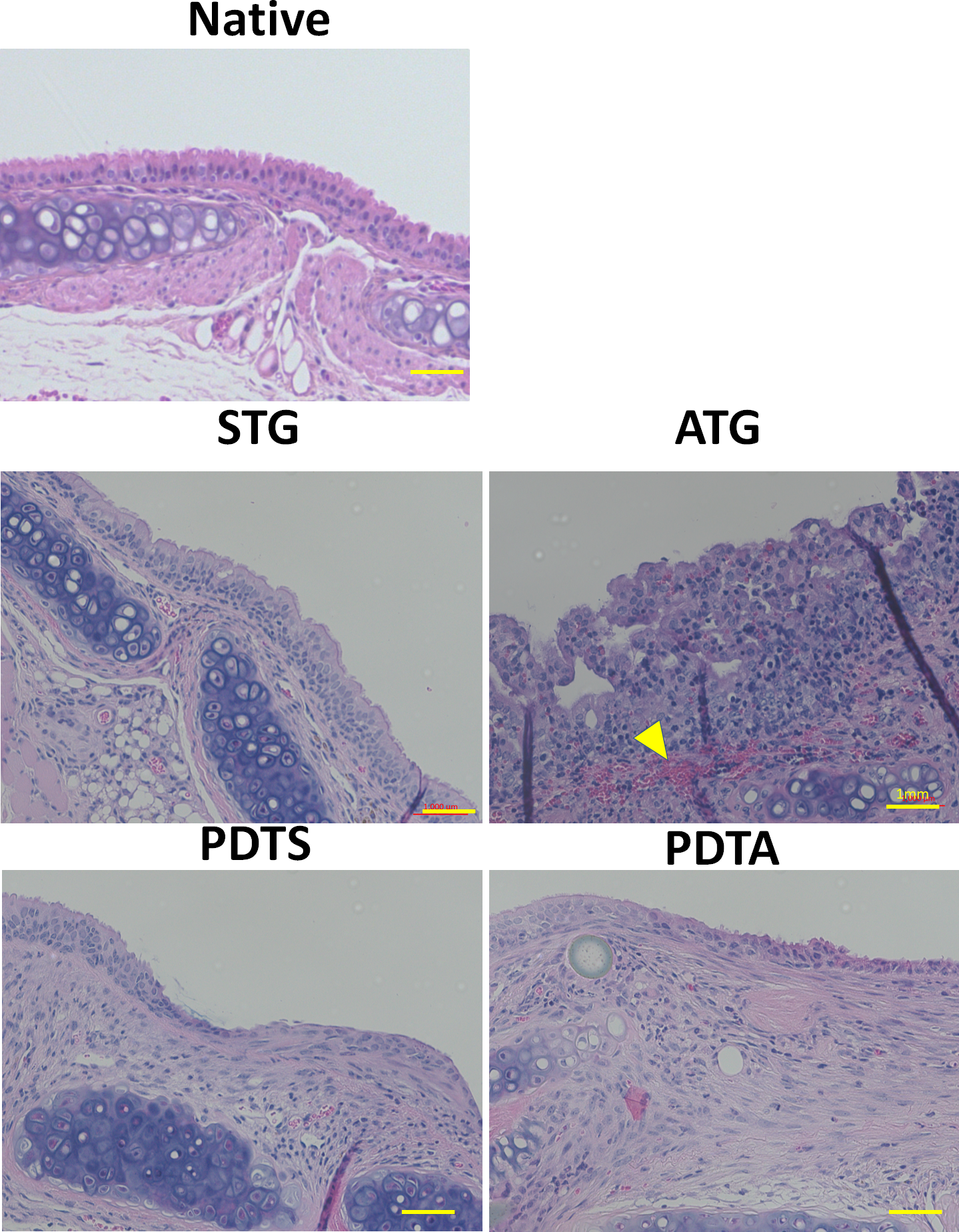

Supplement: Supplementary file 1 — Figure S1: Tracheal allograft presents with epithelial sloughing and eosinophilic infiltrates. Representative images of native trachea and tracheal grafts at 10 days post‐implantation. Native trachea and STG present with a columnar pseudostratified epithelium while ATG present with epithelial sloughing and eosinophilic infiltrates denoted by ▼. PDTS and PDTA presents with ciliated neo‐epithelium repopulating the graft. [file BTM2-8-e10525-s002.tif]

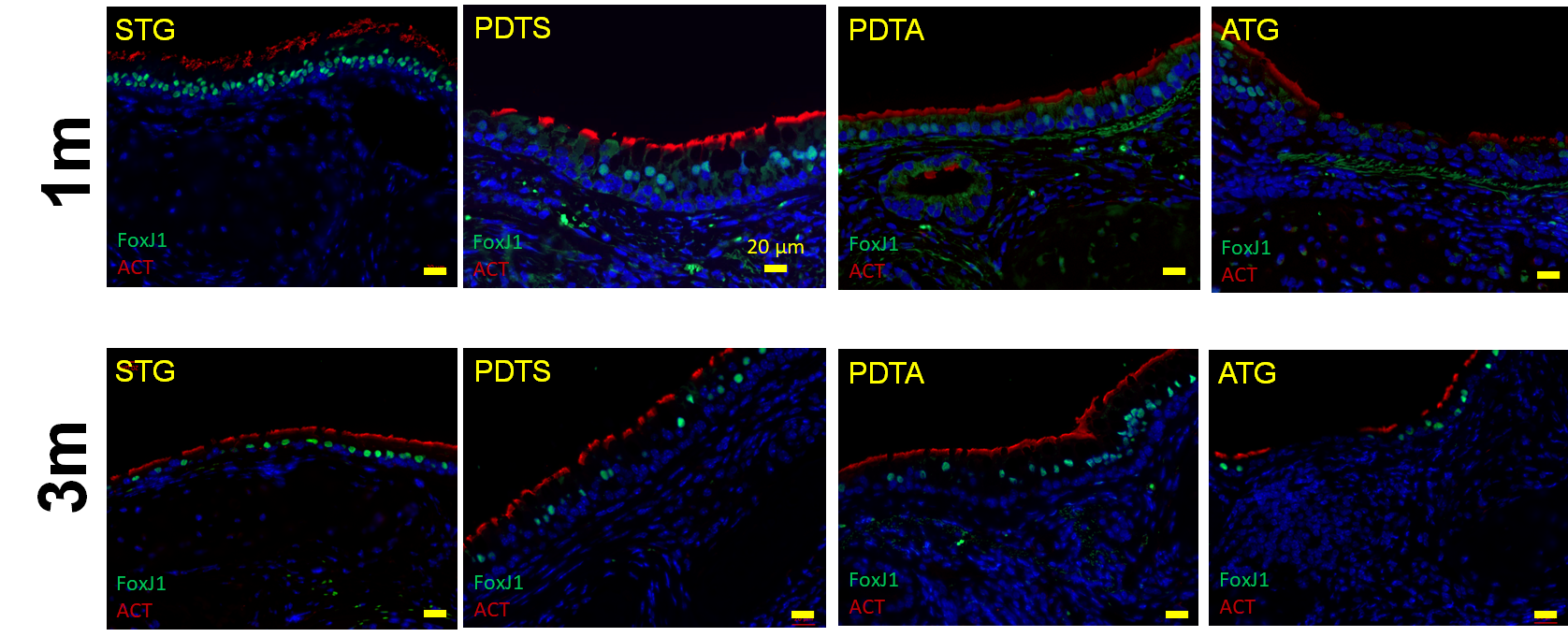

Supplement: Supplementary file 2 — Figure S2: Representative IF images of epithelization (ACT+) for the grafts at 1‐ and 3‐months post implant. Green denotes terminally differentiated ciliated cells (FoxJ1) while red denotes ciliated epithelium (ACT). PDTA and PDTS had similar epithelialization compared to STGs while ATGs had less ciliated epithelium. [file BTM2-8-e10525-s001.tif]

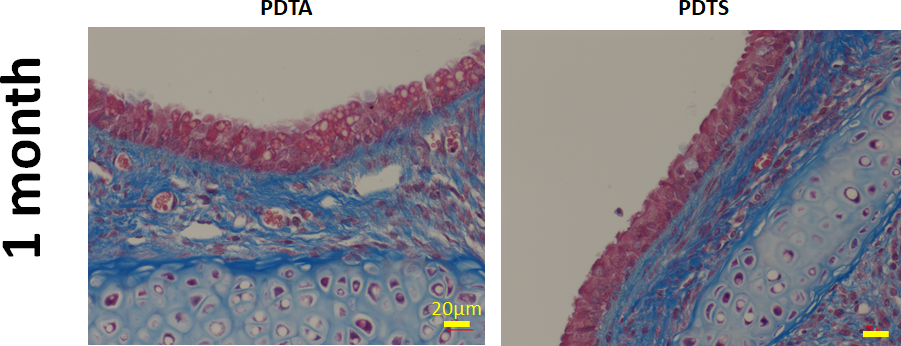

Supplement: Supplementary file 3 — Figure S3: Representative Masson Trichrome stains of PDTA and PDTS at 1 m. Blue indicates collagen deposition. Similar amounts of collagen were observed between PDTA and PDTS, indicating that they are likely to have similar amounts of collagen deposition and no fibrosis as a result from rejection is occurring. [file BTM2-8-e10525-s003.tif]
